# Physical Activity Patterns According to the Type of Physical Education Classes by Sex and Obesity among Korean Adolescents

**DOI:** 10.3390/ijerph20043151

**Published:** 2023-02-10

**Authors:** Gyuil Lee, Seyong Jang, Sunga Kong

**Affiliations:** 1 Department of Physical Education, Teachers College, Kyungpook National University, Daegu 41566, Republic of Korea; 2 Department of Taekwondo, College of Arts and Physical Education, Gachon University, Seongnam 13120, Republic of Korea; 3 Patient-Centered Outcomes Research Institute, Samsung Medical Center, Seoul 06351, Republic of Korea; 4 Department of Clinical Research Design and Evaluation, Samsung Advanced Institute for Health Sciences & Technology, Sungkyunkwan University, Seoul 06351, Republic of Korea

**Keywords:** physical activity, adolescent, game play time, free activity time

## Abstract

This study aimed to compare physical activity patterns according to the type of physical education by sex and body mass index categories among Korean adolescents. We analyzed physical activity using an accelerator in a physical education class among Korean middle school students (1305 boys and 1328 girls). An independent *t*-test and regression analysis were conducted to compare differences between the obesity groups by sex. As game play time increased, light activity increased in boys in the normal group. Among the girls, sedentary time decreased in the normal, at-risk for obesity, and obese groups. Moderate activity increased in the underweight, normal, at-risk for obesity, and obese groups. Vigorous activity increased in the normal group. As free activity time increased, sedentary time also increased in the normal, at-risk for obesity, and obese groups. Vigorous activity decreased in the normal group. Among the girls, sedentary time increased in the underweight group. Light activity decreased in the underweight and normal groups. A strategy to increase physical activity during physical education class is to increase game play time for girls and decrease free activity time for boys.

## 1. Introduction

Obesity is a public health concern in many countries [1]. The prevalence of being overweight or obese among children and adolescents aged 5–19 years has risen sharply from just 4% in 1975 to over 18% in 2016. This increase has been similar in both boys and girls [2]. Obesity during early life remains a leading public health challenge, as it is linked to poor long-term physical and mental health [3].

Physical activity is a healthcare intervention for poor mental and physical health, including problems such as being overweight or obese. Although preventing excess weight and obesity in youth is a public health priority, the quality of physical education classes for physical activity promotion is marginalized in practice. During adolescence, physical education has been identified as a potential area for promoting moderate–vigorous activity [4]. Although the educational characteristics of physical education differ among school systems, the provision of moderate–vigorous activity time for youth during school days appears to be a common factor [5]. Furthermore, interventions have the potential to increase the volume of moderate–vigorous activity time during physical education classes [6], suggesting that regular participation in physical education may result in substantial public health benefits, if appropriately designed. Nevertheless, the difference in obesity among students is assumed to be due to the differences in the level of physical activity stimulation, even in the same physical education class.

In general, in addition to a decrease in physical activity with increasing age, the attitudes of children towards physical activity and physical education differ by grade level and sex [7]. Studies have concentrated on students’ attitudes toward physical education [8] and a wide range of activities, including exercise, fitness, sports, and/or physical education [9,10]. In other words, activities such as physical education differ from everyday physical activities because physical education is often highly structured and focuses on specific learning goals and skills. Similarly, sports are different, as they are often single-gendered, can be team-centric, and primarily focus on competition or displaying elite athletic performance. These contexts allude to specific attitudes toward these activities. Thus, identifying attitudes toward physical education, sports, or fitness as attitudes toward physical activity may lead to misunderstandings [11]. For example, the amount of activity during games and free activities in the traditional teaching content and structure of physical education is not clear. To elucidate how different types of physical education are linked to or have the greatest impact on physical activity intentions and behaviors, an understanding of multiple types of physical education is required [12]. Therefore, this study aimed to compare physical activity patterns according to the type of physical education by sex and obesity among Korean adolescents.

## 2. Materials and Methods

### 2.1. Participants

We analyzed the physical activity patterns of middle school students in Korea during physical education classes. The study was conducted among boys and girls who participated in physical education classes at 11 middle schools in Korea. Of the 2633 participants, 1305 were boys and 1328 were girls. The mean age was 13.77 ± 0.65 years. Obesity group divided body mass index [13], underweight (<18.5 kg/m^2^, 21.72%), normal (18.5−22.9 kg/m^2^, 49.37%), at-risk for obesity (23.0−24.9 kg/m^2^, 10.25%), and obese (≥25.0 kg/m^2^, 18.65%). This retrospective study was approved by the Institutional Review Board of Sungkyunkwan University (SKKU-2022-02-006). The participants’ characteristics are presented in Table 1.

### 2.2. Measurement

Physical activity was measured using an accelerometer (ActiGraph, Pensacola, Co., Pensacola, FL, USA). The 3D accelerometer measurement was performed once per class (45 min), and the students’ heights and weights were pre-entered into the 3D accelerometer, which they wore around their waists before the class began. Their usual activities were maintained during physical education classes, to measure their physical activity. physical education classes comprised health and physical fitness, track and field, gymnastics, invasive sports (i.e., soccer, basketball, and handball), net-type sports (i.e., volleyball and badminton), and field sports (i.e., baseball and t-ball). The class proceeded with an explanation by the teacher, followed by demonstration, task practice, and formative evaluation. A game play is a task activity conducted for content-related learning of students in a sports activity class, and free play is an activity selected and organized by students regardless of class content. The free activities that students mainly participated in were walking, gym exercise, and games (i.e., badminton, soccer, basketball, and foot baseball). The duration of each content in physical education class was different in each class. However, for each class, both game play time and free activity time were recorded, and the provided time for each class was compared through continued values. Classes contained various numbers of obese students, and the same physical education content was applied in each class. However, the data of students who removed their devices during the 45 min classes were excluded, and 12 cases of device errors (e.g., cases where less activity time resulted from issues in uploading the measurement data) were excluded from the data analyses.

### 2.3. Statistical Analyses

Descriptive statistics were used to summarize the characteristics by group. The mean and standard deviation of each of the measured variables were calculated using STATA 15. To analyze the collected data, ActiGraph’s software program Actilife (v6.11.9) was used to store the recorded data on a PC. The participants’ strengths were categorized according to the cutoff points of physical activity intensity proposed by Evenson et al. [14], which have been validated [15] specifically for children: sedentary (≤50 counts), light (51–1148 counts), moderate (1149–2005 counts), and vigorous (≥2006 counts) [14]. An independent *t*-test and chi-square test were performed to compare the differences in characteristics by sex. One-way analysis of variance was performed to compare the differences in the duration of physical activity during physical education class by the obesity group. Linear regression analysis was performed to analyze the relationship between physical activity time and game play time during physical education classes in the obesity group, after adjusted for age. The level of statistical significance was set to *p* < 0.05.

## 3. Results

Among the boys, sedentary physical activity time was the shortest in the obese group (*p* < 0.05). Light activity time was shortest in the overweight and obese groups (*p* < 0.05). There were no significant differences in the moderate and vigorous activity levels between the groups (*p* < 0.05) (Table 2). Among the girls, sedentary time was shorter in the overweight and obese groups than in the normal group (*p* < 005). The light activity time was significantly longer in the obese group than in the underweight and normal groups (*p* < 0.05). Vigorous activity time was the longest in the overweight group and was significantly longer than that in the normal group (*p* < 0.05). The shortest vigorous activity time was observed in the obese group (*p* < 0.05) (Table 2).

Regarding the rate of physical activity by intensity during the physical education class according to obesity, light activity time was the longest in the obese group (*p* < 0.05). 

Among the boys, light activity time was longer in the obese group than in the underweight, normal, and at-risk of obesity groups (*p* < 0.05). However, sedentary, moderate, and vigorous activity proportions were not significantly different among the groups (*p* < 0.05).

**Figure 1:**
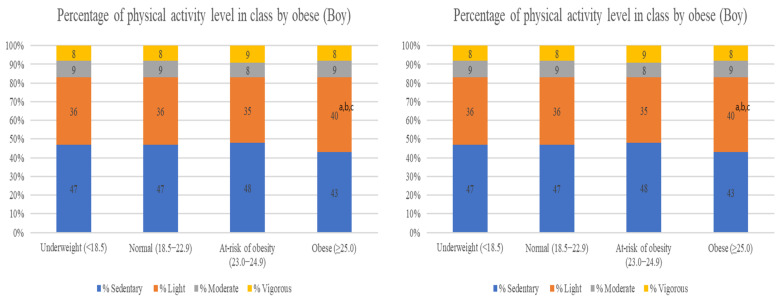
Percentage of physical activity level in class by obese. Data are presented as mean ± standard deviation; one-way analysis of variance. a *p* < 0.05 compared with the underweight group. b *p* < 0.05 compared with the normal group. c *p* < 0.05 compared with the at-risk for obesity group.

Among the girls, sedentary time was shorter and vigorous activity time was longer than that in the at-risk of obesity group (*p* < 0.05). The sedentary time was significantly shorter in the obese group than in the normal group, and the light activity time was the longest (*p* < 0.05) in the obese group than in the underweight and normal groups. However, the vigorous activity time ratio was shorter in the obese group than the at-risk of obesity group (*p* < 0.05) (Figure 1).

Among the boys, the light activity time increased only in the normal group for activity time by intensity according to game play time (*p* < 0.05). No significant differences were observed between the groups (*p* > 0.05) (Table 3).

Among the girls, activity time by intensity according to game play time showed increases in the amounts of moderate and vigorous activity time in the underweight group (*p* < 0.05, respectively); however, sedentary time decreased in the underweight group (*p* < 0.05). In the normal-weight and at-risk for obesity group, sedentary time decreased, and moderate and vigorous activity levels increased (*p* < 0.05, respectively). In the obese groups, the sedentary time decreased; however, moderate activity time increased significantly (*p* < 0.05, respectively) (Table 3).

Among the boys, in the normal group, the sedentary time increased; however, light and moderate activity time decreased significantly (*p* < 0.05). In the at-risk for obesity group, the sedentary time increased; however, the light activity time decreased (*p* < 0.05). In the obese group, the sedentary time increased (*p* < 0.05). In the underweight group, no significant differences were observed between the activity time by intensity according to free activity time (*p* < 0.05) (Table 4).

Among the girls, in the at-risk for obesity group, only the sedentary time increased (*p* < 0.05). In the underweight, normal, and obese groups, no significant differences were observed between the activity time by intensity according to free activity time (*p* < 0.05) (Table 4).

## 4. Discussion

This study confirmed the differences in physical activity patterns according to the type of physical education by sex and obesity among Korean adolescents. Compared to the other groups, light activity time was significantly longer during the physical education class among obese boys. Furthermore, among the obese girls, sedentary time was shorter, and light activity time was the longest, compared to those in the other groups; however, vigorous activity time was the shortest. Depending on the type of physical education class, as game play time increased, sedentary time decreased in girls; and light, moderate, and vigorous physical activity time increased. As free activity time increased, sedentary time increased, while light, moderate, and vigorous activity levels decreased. This trend was stronger among a normal group of boys than it was in a group of girls. For a healthy body through the promotion of physical activity, it would be helpful to increase the game play time for girls and reduce the free play time for boys.

Among adolescents, physical education classes are positively associated with physical activity [16]. In a previous study, compared to those who did not participate in physical education classes, those who participated in physical education classes ≥ 3 days/week had double the odds of being sufficiently active (odds ratio 2.05, 95% confidence interval 1.84–2.28). There were no apparent gender or age group differences [17]. In both sexes, physical education classes were associated with longer physical activity levels [18]. Nevertheless, a previous study demonstrated that normal-weight students were more physically active than overweight and obese students during physical education classes [19]. Therefore, moderate–vigorous activity time may vary significantly according to the type of physical education lesson [13]. Thus, physical education teachers and leaders should develop strategies and programs to help adolescents participate actively in physical education classes.

The greatest benefit of voluntary participation is the enjoyment of the physical activity. In the physical education class, students actively participate in fun activities. Game activities, including sports games, are representative activities that attract students’ interest. Therefore, in general, moderate–vigorous activity time during the physical education class is positively correlated with game activity time [20,21]. Adolescents aged 11–14 years engage in the most moderate-vigorous activity time during team games (e.g., football) and individual activities (e.g., athletics) and the least during individual games (e.g., badminton) and movement activities (e.g., dance) [22]. A similar finding was reported among children aged 5–11 years: higher levels of moderate–vigorous activity performed during team games than during movement activities [23]. Our results showed that physical activity levels during game activities had a positive effect only on girl students. Students learn sports in game play class. Sex differences exist in physical activity participation: generally, there is more effort put in by boy students than among girl students [24]. Accordingly, girl students might have been more stimulated in their physical activity because they had less experience in sports activity compared to boy students. Male students were not affected more in the game play class and became inactive in the free activity class. 

Voluntary participation in physical activity is influenced by the subjective judgment of “Am I able?” rather than “Is it worth it?” [25]. In this respect, programs that promote physical activity should consist of “fun and doable task activities”. The enjoyment of physical activity is positively correlated with physical activity participation levels among children and adolescents [26]. physical education classes have also been identified as predictors of physical activity [27,28]. In the case of free activity, physical activity was negative compared to the game play class. A previous study demonstrated that when the program details are examined, the success rate of the physical-activity-oriented programs appears to be higher in all variables [29]. A physical education program that not only targets positive perceptions of competence, but also provides a variety of different activities, allowing participants to choose those that most interest them, is likely to produce greater levels of intrinsic motivation and enjoyment [30]. However, in our study, game play class encouraged physical activity among girl students. Therefore, greater exposure to game play classes, including territorial competition (i.e., soccer, basketball, and handball), net-type competition (i.e., volleyball, tennis, and badminton), and on-field competition (i.e., baseball, T-ball, and softball) sports activities will help promote physical activity and prevent public health problems of obesity among middle school students, especially girls.

This study had several limitations. First, it is difficult to generalize our findings to adolescents globally because we examined only middle school students in Korea. However, our study analyzed the data of over 1000 students in Korea, giving it relatively high statistical power. Second, we did not include clinical indicators, such as general health, mental health, or disease. Third, because this was a retrospective study, cause-and-effect relationships could not be assessed, and only interrelationships between variables were examined. Lastly, all participants were middle school students; however, matching the number of boys to girls by grade level was challenging. Although the physical education contents of the schools were similar, this study was conducted within a middle school, and proportionality of the sex of the subjects was not achieved. Accelerometers have also been used for this purpose. Accelerometers can accurately detect physical movements [31]. This is novel, because the amount of physical activity by type of physical education class was objectively examined using an accelerometer for more than 1000 adolescents.

## 5. Conclusions

This study examined the differences in physical activity patterns according to the type of physical education class by sex and obesity among Korean adolescents. According to the type of physical education class, as the game time increased, the sedentary time decreased among girls, and the physical activity increased in terms of light, moderate, and vigorous activity levels. As the free activity time increased, the sedentary time increased; and the light, moderate, and vigorous activity times decreased. This trend was stronger among boys than it was among girls. We confirmed the differences in physical activity patterns according to sex and type of physical education class. A strategy to increase physical activity during physical education class is to increase game play time for girls and decrease free activity time for boys.

## Figures and Tables

**Table 1:** Physical characteristics by sex.

	Total (N = 2633)	Boys (n = 1305)	Girls (n = 1328)	*p*-Value
Age (year)	13.77 ± 0.65	13.82 ± 0.65	13.73 ± 0.62	0.002 **
Height (cm)	162.36 ± 7.35	166.40 ± 6.91	158.38 ± 5.31	<0.001 ***
Weight (kg)	61.92 ± 13.99	61.92 ± 13.99	53.02 ± 10.10	<0.001 ***
Body mass index (kg/m^2^)	21.68 ± 4.06	22.27 ± 4.42	21.09 ± 3.58	<0.001 ***
Group (n, %)				
Underweight (<18.5 kg/m^2^)	572 (21.72)	264 (20.23)	308 (23.19)	<0.001 ***
Normal (18.5–22.9 kg/m^2^)	1300 (49.37)	560 (42.91)	740 (55.72)	
At-risk for obesity (23.0–24.9 kg/m^2^)	270 (10.25)	155 (11.88)	115 (8.66)	
Obese (≥25.0 kg/m^2^)	491 (18.65)	326 (24.98)	165 (12.42)	

Values are represented as the number of participants (%) or as the mean ± standard deviation; ** *p* < 0.01, *** *p* < 0.001, independent *t*-test or chi-squared test.

**Table 2:** Time of physical activity in class by obesity group.

	Sedentary	Light	Moderate	Vigorous
Boy				
Underweight (<18.5)	21.30 ± 7.68	16.11 ± 5.19	4.07 ± 2.45	3.48 ± 2.89
Normal (18.5–22.9)	21.13 ± 7.55	16.12 ± 5.27	4.05 ± 2.22	3.67 ± 3.18
At-risk for obesity (23.0–24.9)	21.80 ± 8.74	15.50 ± 5.88 ^b^	3.69 ± 2.05	3.95 ± 3.71
Obese (≥25.0)	19.46 ± 7.71	17.87 ± 5.48 ^abc^	4.14 ± 2.23	3.38 ± 2.57
*p*-value	0.037 *	<0.001 ***	0.380	0.379
Girl				
Underweight (<18.5)	14.72 ± 7.89	17.22 ± 4.54	6.23 ± 3.02	6.80 ± 4.65
Normal (18.5–22.9)	15.13 ± 7.56	17.39 ± 4.74	6.11 ± 2.89	6.31 ± 4.15
At-risk for obesity (23.0–24.9)	13.22 ± 7.03 ^b^	17.71 ± 4.87	6.59 ± 3.22	7.40 ± 4.83 ^b^
Obese (≥25.0)	13.58 ± 7.38 ^b^	19.00 ± 5.15 ^ab^	6.32 ± 3.00	6.03 ± 3.91 ^c^
*p*-value	0.004 **	<0.001 ***	0.341	0.004 **

Data are presented as the mean ± standard deviation; * *p* < 0.05, ** *p* < 0.01, *** *p* < 0.001, one-way analysis of variance. ^a^ *p* < 0.05 compared with the underweight group. ^b^ *p* < 0.05 compared with the normal group. ^c^ *p* < 0.05 compared with the at-risk for obesity group.

**Table 3:** Differences in physical activity time by game play time in physical education class for each obesity group.

	Sedentary	Light	Moderate	Vigorous
Boy				
Underweight (<18.5)	−0.039 (−0.129–0.050)	0.038 (−0.022–0.098)	0.015 (−0.014–0.043)	−0.019 (−0.051–0.014)
Normal (18.5−22.9)	−0.046 (−0.101–0.008)	0.041 * (0.003–0.078)	0.009 (−0.008–0.026)	−0.010 (−0.028–−0.009)
At-risk for obesity (23.0−24.9)	0.005 (−0.170–0.180)	0.019 (−0.097–0.135)	−0.006 (−0.046–0.034)	−0.024 (−0.093–0.045)
Obese (≥25.0)	−0.066 (−0.193–0.061)	0.071 (−0.026–0.169)	−0.012 (−0.050–0.026)	−0.012 (−0.056–0.032)
Girl				
Underweight (<18.5)	−0.135 * (−0.227–−0.442)	0.007 (−0.048–0.063)	0.059 * (0.020–0.099)	0.064 * (0.002–0.128)
Normal (18.5−22.9)	−0.142 * (−0.199–−0.084)	0.009 (−0.027–0.046)	0.065 * (0.042–0.087)	0.061 * (0.028–0.094)
At−risk for obesity (23.0−24.9)	−0.137 * (−0.231–−0.043)	−0.022 (−0.096–0.052)	0.085 * (0.036–0.134)	0.068 * (0.001–0.135)
Obese (≥25.0)	−0.143 * (−0.228–−0.058)	0.032 (−0.032–0.095)	0.074 * (0.041–0.106)	0.030 (−0.015–0.075)

* *p* < 0.05; linear regression analysis after adjusted for age.

**Table 4:** Differences in physical activity time by free activity time in physical education class for each obesity group.

	Sedentary	Light	Moderate	Vigorous
Boys				
Underweight (<18.5)	0.075 (−0.044–0.195)	−0.053 (−0.133–0.027)	−0.030 (−0.067–−0.008)	−0.011 (−0.033–0.054)
Normal (18.5−22.9)	0.181 * (0.091–0.270)	−0.113 * (−0.175–−0.050)	−0.042 * (−0.070–−0.014)	−0.024 (−0.055–−0.008)
At-risk for obesity (23.0−24.9)	0.270 * (0.040–0.500)	−0.216 * (−0.366–−0.066)	−0.051 (−0.104–−0.002)	−0.001 (−0.093–0.095)
Obese (≥25.0)	0.056 * (−0.145–0.256)	−0.064 (−0.219–0.091)	−0.029 (−0.089–0.030)	0.045 (−0.023–0.114)
Girls				
Underweight (<18.5)	−0.048 (−0.208–0.112)	−0.085 (−0.180–0.009)	0.039 (−0.030–0.108)	−0.097 (−0.012–0.205)
Normal (18.5−22.9)	0.002 (−0.092–0.095)	−0.056 (−0.115–0.003)	0.020 (−0.017–0.058)	−0.039 (−0.014–0.092)
At−risk for obesity (23.0−24.9)	−0.180 * (−0.350–−0.010)	0.088 (−0.044–0.218)	0.065 (−0.026–0.156)	0.035 (−0.087–0.156)
Obese (≥25.0)	−0.024 (−0.141–0.093)	−0.034 (−0.120–0.052)	−0.018 (−0.028–0.064)	−0.043 (−0.018–0.104)

* *p* < 0.05; linear regression analysis after adjusted for age.

## Data Availability

The datasets used and/or analyzed in the current study are available upon reasonable request from the corresponding author (Sunga Kong).
